# Physicochemical and Functional Evaluation of Chia Mucilage (*Salvia hispanica*)–Alginate Microcapsules as a Delivery System of ACE-Inhibitory Peptides from *Phaseolus lunatus*

**DOI:** 10.3390/plants15050704

**Published:** 2026-02-26

**Authors:** Valentino Mukthar Sandoval-Peraza, David Betancur-Ancona, Arturo Castellanos-Ruelas, Yossef Hernández-Rodríguez, Luis Chel-Guerrero

**Affiliations:** 1 Escuela de Ciencias de La Salud, Universidad Del Valle de México, Calle 79 No 500 Col. Dzityá, Altura Km 9.5 de La Carretera a Progreso, Mérida 97302, Yucatán, Mexico; valentino_sandoval@my.uvm.edu.mx; 2 Facultad de Ingeniería Química, Universidad Autónoma de Yucatán, Periférico Norte Km. 33.5, Tablaje Catastral 13615, Colonia Chuburná de Hidalgo Inn, Mérida 97203, Yucatán, Mexico; bancona@correo.uady.mx (D.B.-A.); cruelas@correo.uady.mx (A.C.-R.); a23215799@alumno.uady.mx (Y.H.-R.)

**Keywords:** antihypertensive activity, bioactive peptides, controlled release, ionic gelation, remanent bioactivity, protective coats

## Abstract

Biopolymers and bioactive peptides of plant origin represent sustainable resources with high potential for the development of functional ingredients with health benefits. An underutilized plant source of antihypertensive peptides is lima bean protein (*Phaseolus lunatus*); however, these peptides can be inactivated or degraded during their passage through the gastrointestinal tract. This study evaluated chia (*Salvia hispanica*) mucilage (CM) combined with sodium alginate (Al) as a hybrid encapsulation matrix for ACE-inhibitory peptides (<10 kDa) from *P. lunatus*. The ionic gelation technique was used, and encapsulation conditions were optimized using a 2^3^ factorial design that evaluated CM:Al ratios, calcium concentration, and hardening time. The optimal formulation (30:70 CM:Al; 0.05 M CaCl_2_; 20 min of hardening time) achieved approximately 48% encapsulation efficiency and maintained the peptides’ ACE-inhibitory (IC_50_ mg/mL) activity during simulated gastric digestion with controlled intestinal release. The formed capsules demonstrated good flow properties, thermal stability up to 178 °C, and preserved ACE-I activity (0.1 mg/mL IC_50_) significantly better than alginate alone after in vitro digestion. These findings suggest that CM:Al blends could produce capsules with the ability to protect bioactive peptides with low molecular weight, warranting further investigation through in vivo bioavailability studies and structural characterization to confirm the proposed matrix-enhancing mechanisms.

## 1. Introduction

According to the World Health Organization, cardiovascular diseases continue to be one of the leading causes of mortality in the global population to date. It has been reported that around 33% of the world’s population aged between 39 and 79 (1.4 billion people) suffers from hypertension, which is characterized by high blood pressure in the blood vessels exceeding 140/90 mmHg. In most cases, the person shows no signs of the condition [1]. The main recommendations for controlling it are changes related to diet and lifestyle, such as reducing salt intake, implementing a moderate exercise program, and following a heart-healthy eating pattern (such as the DASH diet, which is a dietary approach to stopping hypertension) [2]. An increase of 50% in people using hypertension management has been proposed to prevent approximately 76 million deaths between 2023 and 2050 [1].

One of the mechanisms for quantifying antihypertensive activity is by measuring the inhibition of angiotensin-converting enzyme 1 (ACE-I), which reduces the conversion of angiotensin I to angiotensin II (a vasoactive peptide). This in turn binds to blood vessel receptors, causing them to contract, increasing blood pressure [3]. Drugs like hydrochlorothiazide, captopril, losartan, metoprolol, and nifedipine are commonly used and highly effective against hypertension, but they can produce adverse effects such as dry cough, edema, palpitations, headache, rapid pulse, dyspnea, dizziness, and impaired renal function with prolonged use [4]. Because of these adverse effects, there has been a recent increase in the promotion and use of health-promoting compounds derived from natural sources for possible management of hypertension. In this regard, antihypertensive peptides derived from plant proteins are produced when hydrolysis generates specific chains of amino acids with biological activity [5]. It has been reported that hydrolysis with enzymes such as papain, pepsin, and trypsin generates peptides with remarkable ACE inhibition [6]. In addition, different researchers mention that they have low toxicity and are easily metabolized by the body [4], but the physiological relevance must be validated through in vivo research to guarantee this.

A promising plant source for obtaining peptides with antihypertensive activity is the beans of the *Phaseolus lunatus* legume. Peptides with ACE-I activity obtained using the sequential pepsin–pancreatin enzyme system at different enzyme/substrate levels, protein content, and hydrolysis times have been reported, generating peptides with ACE-I activities in the range of 0.25–0.69 mg/mL IC_50_ [7]. This range of activity is similar to reports for other legume hydrolysates like *Cicer arietinum*, *Phaseolus vulgaris*, *Lens culinaris*, *Pisum sativum*, and *Glycine max* (0.673, 0.633, 0.606, 0.595, and 0.224 mg/mL IC_50_, respectively) [8].

Regardless of the source of the hydrolysates and their peptides, there are some factors to consider: First, the size of the peptides present in the hydrolysates, since those with a molecular weight of ≤3 kDa have been reported to have better antihypertensive activity, proposing the ultrafiltration process to separate protein hydrolysates into peptides of different molecular weights [9]. Second, the enzymes used to produce the hydrolysates—for example, trypsin, which cleaves protein with Lys and Arg residues in the carboxyl side; α-chymotrypsin, which releases peptides with Ala, His, Leu, Phe, Pro, Tyr, and Val C-terminal amino acids [10]; and pepsin, which cleaves after Phe, Leu, and rarely His and Lys, unless they are next to Leu, Phe, or others [11], are known for their ACE-inhibitory capacity. Lastly, the effect of the amino acid and its sequence in the ACE-I; for example, peptides with high content of Asp and Glu (acidic amino acids) have a negative charge that can chelate zinc atoms, which are needed for the angiotensin enzyme for its activity [12]; peptides rich in amino acids like hydrophobic residues (Ile, Leu, Phe, Pro, and Val), aromatic (Tyr and Trp), and positive charge (Lys and Arg) have a strong influence on ACE binding [5].

Despite these benefits, there are intrinsic properties of peptides that may limit their use, such as chemical degradation, low solubility, hygroscopicity, bitter taste, and, particularly, low resistance to the action of digestive enzymes. For this reason, encapsulation may be a promising method for improving stability, protecting bioactivity and functionality, and controlling peptide release [13]. Ionic gelation is a widely used encapsulation technique due to its mild processing conditions; since it does not involve organic solvents or high temperatures [14], this method effectively preserves the structural integrity and biological activity of sensitive peptides. Sodium alginate is a natural polysaccharide that is used in this technique due to its chemical structure, biocompatibility, safety for the human body, and biodegradability. As an encapsulating material, it has proven to be a safe and effective drug delivery system [15]. Another polysaccharide that has recently been under study is chia mucilage (CM) due to its technological properties, as it has exceptional water retention, gel-forming properties, viscosity reduction, and emulsion stabilization [16]. In addition, it is a source of polysaccharides composed mainly of xylose, arabinose, glucose, galactose, and glucuronic and galacturonic acids [17]; this composition positions CM as a natural plant material with high potential to produce edible coatings [16]. In 2024, microencapsulation technology constituted approximately 52% of global encapsulated food production, corresponding to 101,000 metric tons. The global food encapsulation market is estimated to reach US$ 43,306.02 million in 2026 and US$ 72,314.1 million by 2035 [18]. Beyond the technological progress in encapsulation, increasing attention is being directed toward sustainable materials with a low environmental impact. In this context, mucilage extracted from plant sources has gained interest as a renewable biopolymer with strong potential for application in food systems aimed at satisfying the growing demand for sustainable and health-oriented products among a rapidly expanding population [19].

Despite growing interest in plant-derived peptides with ACE-I activity, these may be susceptible to degradation by gastrointestinal proteolysis, which would limit their bioavailability and biological effect. Therefore, to preserve and protect their biological properties, techniques such as ionic gelation encapsulation have been proposed, as this method preserves the bioactivity of peptides by avoiding the use of solvents or thermal processes that could inactivate them. Chia mucilage has functional properties that make it suitable for use in the ionic gelation encapsulation of essential oils [20] and microorganisms such as lactobacilli [21]; however, its use for the encapsulation of peptides with biological activity has not been reported. Previous studies of underutilized mucilages, such as those from *Delonix regia* [22] or *Guazuma ulmifolia* [23], combined with alginate, have demonstrated effective protection of the bioactivity of plant-derived peptides. For this reason, it is hypothesized that the incorporation of chia mucilage into alginate matrices will promote peptide retention, as these combinations have been shown to improve the gel strength, morphology, and swelling properties of microparticles [24]. The objective of this study was to develop and optimize a hybrid encapsulation system based on chia mucilage and sodium alginate that protects ACE-inhibiting peptides (<10 kDa) derived from *P. lunatus*, ensuring their stability and preservation of bioactivity during simulated gastrointestinal digestion in comparison with pure alginate.

## 2. Results

### 2.1. Proximate Composition of the Raw Material

Table 1 shows the obtained values for the proximal composition of whole chia seed flour (CF) the extracted mucilage (CM), and whole *P. lunatus* bean flour (PF) and its protein concentrate (PPC).

For the CM, it was observed that the largest constituent was carbohydrates, expressed as NFE (68.13%), followed by crude fiber (16.64%). It is in these components that the polysaccharides responsible for the techno-functional activities attributed to mucilage, such as water retention and gel formation, among others, are found. The PPC was obtained by precipitation at the isoelectric point, with a final protein content of 70.36%, achieving a concentration of proteins relative to those present in PF. This is desirable, since the higher the protein content, the greater the possibility of generating hydrolysates or peptide fractions with biological activity.

### 2.2. Degree of Hydrolysis and Initial ACE-I Activity

The degree of hydrolysis obtained from PPC after 90 min of reaction with the sequential pepsin–pancreatin system was 25%. This hydrolysate (PH) had a protein content of 54.77%, and the peptide fraction of <10 kDa obtained by ultrafiltration had a protein level of 49.48% (4.42 mg/mL). Fractionation by molecular weight was performed based on previous studies, which showed that, particularly for *P. lunatus* hydrolysates, the smaller-molecular-weight fractions tend to concentrate the peptides responsible for ACE inhibition. Consequently, the <10 kDa fraction was selected for the encapsulation process. ACE inhibition assays confirmed this trend: the peptide fraction (<10 kDa) showed greater inhibitory potency compared to the hydrolysate (0.38 and 0.73 mg protein/mL IC_50_, respectively). These values indicate that ultrafiltration allowed the bioactive peptides to be concentrated, approximately doubling the inhibitory activity.

### 2.3. Shape, Morphology, and Diameter of the Capsules

Table 2 shows the diameters and shapes of the capsules obtained with the treatments and their respective controls (without peptide fraction). All the capsules formed were irregularly hemispherical. Statistically, no significant differences (*p* > 0.5) were found between the controls and the treatments; however, it can be observed that the capsules with peptide fraction showed a slight increase in area. The final morphology of the capsule along with the particle size are important parameters, as they allow us to predict whether they will move well through the digestive tract. Therefore, the smaller the size and the fewer irregularities, the better the flow behavior would be expected to be.

### 2.4. Encapsulation Efficiency

The capsules formed without the peptide fraction according to the experimental design (Table 3) had an initial total protein content of 0.73–1.7%. In the case of the capsules with the peptide fraction, the final content ranged from 9.22% to 14.20%. Treatment 1 had the highest total protein content (12.46%), compared to treatment 7, which had the lowest content (7.70%). Regarding encapsulation efficiency, statistically significant differences (*p* < 0.05) were observed, with treatment 1 (30:70 CM:Al; 0.05 M CaCl_2_; and 20 min of hardening) being the most efficient (47.78%), followed by treatment 8 with high levels in its factors (70:30 CM:Al; 0.15 M CaCl_2_; and 30 min of hardening), with an efficiency of 46.19%. This shows that efficiency depends on experimental design factors, the CM:alginate ratio, calcium chloride concentration, and setting time. Regarding alginate control, statistically significant differences (*p* < 0.05) were observed for all treatments, with the CM:Al combination improving encapsulation efficiency.

### 2.5. Calcium Uptake, Angle of Repose, and Differential Scanning Calorimetry (DSC)

Table 4 shows the calcium uptake values during capsule formation. The capsules formed without the peptide fraction show statistically significant differences (*p* < 0.05) in calcium uptake. Statistically significant differences (*p* < 0.05) were found in the capsules to which the peptide fraction was added compared to their blank, which can be attributed to the need for greater calcium ion uptake during ionic gelation in order to trap the fraction inside the particle. Treatment with the highest levels in the design (70:30 CM:Al; 0.15 M CaCl_2_; 30 min of hardening) had a calcium uptake similar to the treatment with the lowest levels of the design (30:70 CM:Al; 0.05 M CaCl_2_; 20 min of hardening) (45.64 and 45.89%, respectively).

The classification used to determine the flow of a capsule in the digestive tract will depend on the angle of repose formed during aggregation, ranging from ‘excellent’ (25–30°) to ‘very, very poor’ (66–90°). Statistically significant differences (*p* < 0.05) were found for this variable, with treatments ranging from a rating of ‘excellent’ (treatment 8) to ‘passable’ (treatment 3). DSC analysis indicated that there were no significant differences (*p* > 0.05) between the thermal transitions of all treatments in this design; however, a slight increase in temperature was observed for the capsules with the peptide fraction.

### 2.6. In Vitro Release, Protein Released, and Remaining ACE-I

The values for the protein released during *in vitro* digestion are shown in Table 5. Statistically significant differences (*p* < 0.05) were observed in the release of the protein fraction under gastric and intestinal conditions. In general, the greatest release occurred under gastric conditions, where the conditions with the highest peptide fraction retention were in the central treatment (CM:Al 50:50; 0.1 M CaCl_2_; 25 min of hardening); however, it had the lowest encapsulation efficiency (33.23%). The best release of the peptide fraction in the intestinal medium was with CM:Al 30:70, 0.05 M CaCl_2_, and 30 min of hardening, with 7.9 mg of protein. To achieve better retention in gastric conditions and greater release in the intestinal environment, not only is the combination of CM:Al, the concentration of the gelling ion, and the hardening time important, but the encapsulation efficiency of the treatment must also be considered. Capsules formed with alginate alone showed statistically significant differences (*p* < 0.05), as peptide fraction retention was not achieved, with 100% of the peptide fraction being released under gastric conditions. This represents an advantage in the use of CM, since at any of its addition levels (30 [*−*], 50 [0], or 70 [+] ratio), the peptide fraction that could be released under intestinal conditions was maintained, thus ensuring that the biologically active component reaches the site where it can exert its bioactivity and the organism can benefit from its effects.

Regarding ACE-I (IC_50_ mg/mL) in gastric medium, statistically significant differences were observed (*p* < 0.05). The central treatment had the highest IC_50_ ACE-I activity (0.15 mg/mL). In the intestinal medium, the best ACE-I (IC_50_) treatments were 3 and 8, both with an 0.06 mg/mL. It is possible that ACE-I is more closely associated with the molecular weight of the peptide fraction than with the quantity, since treatments 3 and 8 had the lowest release under intestinal conditions (3.81 mg) but the best ACE-I activity.

### 2.7. Amino Acid Composition in the <10 kDa Fraction and Released Fraction After Simulated Digestion

The amino acid composition of the <10 kDa peptide fraction obtained from *P. lunatus* is shown in Table 6, where the presence of all amino acids was identified. Usually, hydrophobic amino acids such as Val, Trp, Ile, Phe, Met, Tyr, and Ala, proline, or positively charged amino acids such as Lys and Arg show ACE-I activity. These were found in a concentration of 50.89% in the <10 kDa fraction of *P. lunatus*, observing that the presence of these amino acids could be responsible for the initial ACE-I activity (0.379 mg/mL of IC_50_). In the amino acid profile of the peptides released in the intestinal medium simulation, Trp and Cys were identifiable, but due to their low concentration, they could not be quantified. Val, Ile, Phe, Met, Tyr, Ala, Pro, Lys, and Arg were present in the peptides released from all treatments at a concentration of around 50%. These amino acids may be directly related to the activity range of ACE-I (0.06–0.13 mg/mL IC_50_) quantified in the peptides released into the intestinal medium.

### 2.8. Capsule Formation, Morphology, and Diameter

**Figure 1:**
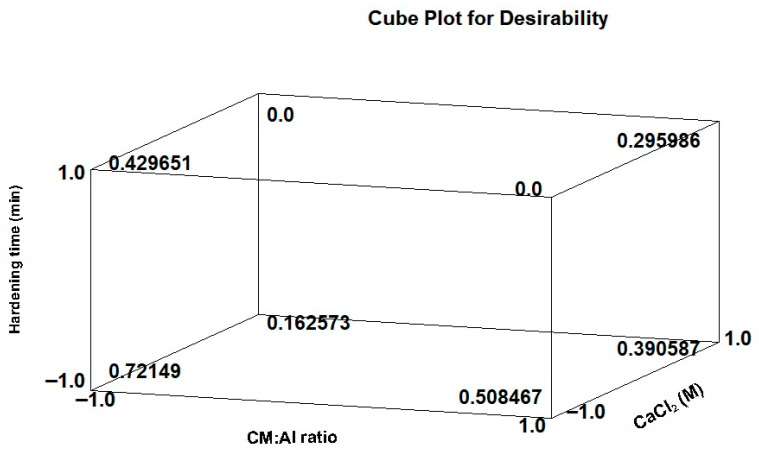
Desirability as optimization of multiple response variables of encapsulated peptide fraction < 10 kDa of *P. lunatus*.

A desirability score (D) with the aim of optimizing ACE-I activity (Figure 1) was calculated for all responses. In accordance with the statistical methodology, all responses were assigned a weighting value of 1 and an impact value (range 1 to 5) based on the effect of the response variable. These values were combined to calculate the composite desirability, where a value of 1 is optimal. Based on the D score (0.72149), the best encapsulation conditions for the peptide fraction < 10 kDa with adequate ACE-I activity are a concentration of CM:Al 30:70 ratio, 0.05 M CaCl_2_, and a hardening time of 20 min. obtaining an encapsulation efficiency of 47.78%, an angle of repose of 35.99, release of 6.40 mg of protein in the intestinal medium, and an I-ACE activity of 0.1 mg of protein/mL of IC_50_.

## 3. Discussion

### 3.1. Proximate Composition of the Raw Material

The main component of CM (Table 1) was carbohydrates, estimated as NFE at 68.13%, followed by crude fiber (16.46%), in which the polysaccharides that compose it were found. The NFE value is comparable to that reported by Santana et al. [25], 68.22%, and it is important to consider this content because there is wide variation in the chemical composition of CM that can be attributed to the place where it is harvested, the seed variety, and soil quality, among other variables [17]. Another factor that has an influence is the method of mucilage extraction, such as seed separation and drying, which can influence the final characteristics of the capsules formed with it [26].

Protein concentrates with a content greater than 65% can commonly be obtained when the protein content in the legume seeds ranges from 21 to 26% [27]. In the case of PF, this content was 21.98%, obtaining a PPC with 70.36% protein. This value indicates that the extraction and concentration process was highly efficient, likely due to the favorable solubility and precipitation of *P. lunatus* proteins. More importantly, the high protein purity of PPC enhances its suitability as a substrate for enzymatic hydrolysis, facilitating the generation of low-molecular-weight peptides with biological activity, particularly ACE-I. Although synthetic ACE inhibitors remain the clinical standard for acute treatment, bioactive peptides may be suitable for preventive strategies and early stages of the disease [28]; however, to achieve this, in vivo and clinical studies must be conducted to ensure their effectiveness. In the case of CM, it was observed that the polysaccharide content is 84.59%, which is responsible for most of its functionality. Furthermore, this plant species is widely distributed and easily accessible, and the mucilage is extracted simply by hydration and filtration [16], making it easy, quick, and inexpensive to obtain [19].

### 3.2. Degree of Hydrolysis and Initial ACE-I Activity

The protein concentrates from *P. lunatus* reached a degree of hydrolysis (DH) of 25% after 90 min of sequential reaction with the pepsin–pancreatin system. This level of hydrolysis suggests that a substantial proportion of the original protein was hydrolyzed; this is important because the generation of bioactive peptides depends strongly on the extent of proteolysis, as higher DH promotes the release of peptides with biological activity [29].

The peptide fraction <10 kDa showed significantly higher ACE-inhibitory activity (IC_50_ = 0.38 mg/mL) compared to the total hydrolysate (IC_50_ = 0.73 mg/mL). This increase in biological potency following fractionation by molecular weight has been previously reported in *P. lunatus*, suggesting that the removal of larger protein fragments reduces steric hindrance at the active site of the angiotensin-converting enzyme (ACE-I) [22,23]. According to Della Rosa et al. [30], when DH levels are high, a large amount of small peptides are generated, and the quantified ACE-I activity could be determined by the amino acid composition of the <10 kDa fraction. The analysis identified a concentration of 50.89% of the key amino acids for enzyme inhibition (Table 6), including hydrophobic residues (Val, Ile, Phe, Met, Tyr, and Ala), aromatic residues (Trp, Phe, and Tyr), and positively charged amino acids (Lys and Arg). According to Tawalbeh et al. [31], the presence of these specific amino acids in the peptide sequences is decisive for the binding affinity with ACE-I, which explains the initial biological activity observed before encapsulation. The results of this study show that ACE-I may have been influenced by the molecular weight fractionation [9] and the presence of amino acids with antihypertensive activity [5,12,31]; however, peptide sequencing is needed to determine the size and specific sequence of the peptides responsible for this activity.

### 3.3. Encapsulation Efficiency and DSC

The experimental factors were systematically adjusted to identify the optimal encapsulation conditions and were selected based on their previously studied influence [22,23]. The encapsulation efficiency obtained under optimized conditions (30:70 CM:Al; 0.05 CaCl_2_; 20 min of hardening time) was 47.78% (Table 3), indicating that this formulation provides a matrix with sufficient structural integrity to retain the peptide fraction (<10 kDa). This efficiency suggests that the combination of CM and Al promotes intermolecular interactions that favor peptide entrapment in the capsule. The efficiency value falls in the range reported for alginate-based systems encapsulating bioactive peptide fractions with the same weight and source (*P. lunatus*) using mucilages from *D. regia* [22] and *G. ulmifolia* [23] with an efficiency of ~31–36% and ~7–17%, respectively. More broadly, the encapsulation efficiency obtained in this study is consistent with values reported for peptide encapsulation by ionic gelation in plant-derived polysaccharide matrices, which typically range from 35 to 65% [14].

The encapsulation efficiency obtained (~48%) can be considered moderate compared to values reported for CM encapsulation of essential oils [20], hydrolysates [32], and colorants [33] (~89, 77.24, and ~95%, respectively). However, these comparisons should be interpreted with caution, as encapsulation efficiency depends on the physicochemical nature of the encapsulated compound and its molecular weight, polarity, and diffusion capacity, as well as on the encapsulation technique used [34]. In particular, the peptide loss during hardening can be principally attributed to three current mechanisms: outward diffusion of soluble peptides through the partially formed gel shell into the large-volume CaCl_2_ solution, before the crosslinking is complete [35]; the high aqueous solubility of low-molecular-weight peptides (<10 kDa), which promotes migration toward the hyperosmotic hardening medium [34]; and transient pores generated by incomplete surface crosslinking during the initial moments of bead formation [36]. These mechanisms are consistent with those reported for other alginate-based systems incorporating plant protein hydrolysates [14]. In this study, ionic gelation was selected to preserve the biological activity of the peptide fraction < 10 kDa, avoiding thermal processes that could compromise its functionality. Under optimal treatment conditions, the incorporation of CM improved peptide retention compared to when alginate alone was used. Although encapsulation efficiency is better for hydrophobic or macromolecular cores, the proposed system represents an effective strategy for protecting low-molecular-weight bioactive peptides and also lays the groundwork for finding conditions that improve trapping efficiency.

An increase in thermal transition temperature (178.68 °C) was observed with DSC in the capsules with peptides. These results may be due to the rich content of xylose, arabinose, and uronic acids in chia mucilage, which may act as a filling agent within the egg-box-like network of alginate, limiting the leaching of low-molecular-weight peptides, in analogy with chitosan–alginate systems that reduce macroporosity [37]. The improvement in bead structural integrity when CM was combined with sodium alginate may be partially related to a complementary space-filling effect within the egg-box network. It is tentatively proposed, pending further structural characterization (e.g., SEM or mercury porosimeter), that CM polysaccharides could occupy interstitial spaces within the gel matrix, potentially reducing effective pore size. This hypothesis is consistent with the thermal (DSC) and morphological observations reported herein; however, it should be regarded as a mechanistic interpretation rather than a confirmed finding until direct structural evidence is available.

### 3.4. Morphology, Area, and Angle of Repose

The capsules obtained from the best treatment had an irregular hemispherical morphology with an area of 5.37 mm^2^. The use of non-conventional mucilage polysaccharides has been associated with heterogeneous polymer distribution and variations in viscosity due to their differences in their molecular composition, which can interfere with the uniform diffusion of Ca^2+^ ions and lead to irregular particle shapes, as previously reported for West Indian elm (*Guazuma ulmifolia*) [23] or flamboyant (*Delonix regia*) [22]. Despite this morphological heterogeneity, the particles displayed favorable flow behavior, with an angle of 35.99°, classified as ‘good’ according to the flow properties scale. This value is indicative of the friction and resistance to movement that the particle would encounter as it passes through the digestive tract. In the case of the capsules under study, there would be less aggregation and friction, resulting in better release of the contents [38]. The angle of repose of the capsules formed solely with alginate was 35.08°, obtaining the same ‘good’ rating as the selected treatment, meaning that the use of CM in combination with Al as a coating material does not affect this parameter.

### 3.5. Protein Released, Residual ACE-I, and Amino Acid Profile

The CM:Al system demonstrated superior intestinal protection compared to pure alginate. The alginate capsules released 100% of their contents into the gastric environment due to their intrinsic porosity. The mixture with CM allowed the protein fraction to be retained for controlled release in the intestine (6.4 mg). This retention is essential to prevent premature chemical and proteolytic degradation of the peptides. It should also be noted that when oral dosing of peptide fractions with more than 20 amino acids is considered, protection during gastrointestinal transit is expected, avoiding proteolytic and chemical degradation and thus ensuring that the peptide reaches a specific area and exerts its bioactivity [39]. The retention in the gastric environment does not mean better bioavailability; this has been reported for peptides administered orally through tablets in monkeys, where retention of the peptide in the gastric phase was reported, but this did not translate to greater activity [40]. Therefore, CM shows high potential for encapsulation, as it withstood gastrointestinal digestion and maintained the ACE-I activity of the <10 kDa peptide fraction of *P. lunatus*; however, intestinal absorption, epithelial transport or cell models, and in vivo studies should be carried out to understand the real bioavailability.

Tawalbeh et al. [31] reported that if a peptide fraction contains amino acids such as Cys, Pro, Leu, and Met (hydrophobic); Trp, Phe, and Tyr (aromatic); Arg and Lys (positively charged); and Val and Ile (branched-chain aliphatic), it will have ACE-I activity. All the aforementioned amino acids, excepting Cys and Trp, were present in the <10 kDa peptide fraction released into the intestinal medium by treatment 1, with a percentage of presence of 55.68%, which would explain its ACE-I activity of 0.1 mg/mL; however, ACE-I activity is ultimately governed by specific peptide sequences and structural motifs rather than by overall amino acid composition alone. Further identification of individual peptides by LC-MS/MS and in silico docking studies would be necessary to confirm which specific sequences are responsible for the observed ACE-I activity. The inhibition value of treatment 1 was higher than that reported for legume hydrolysates such as soybean and lupin (0.224 and 0.226 mg/mL IC_50_) [8]. The ACE-I value in this study was also higher than that reported by Senadheera et al. [41] for hydrolysates from animal sources such as salmon skin collagen, cuttlefish hydrolysates, and goby fish protein hydrolysates with an IC_50_ of 1.165, 1.58, and 1.36 mg/mL.

The findings of this study show that CM is a plant-based source with high potential for use in the encapsulation of peptide fractions for various reasons. First, the seeds are widely available, as they can be found all year round. Second, CM extraction is simple, as it only involves hydrating the seed at room temperature, resulting in CM with encapsulating properties. Third, all capsules formed under experimental conditions (Table 7) achieved protection of the peptide fraction with ACE-I activity during the simulated digestive process compared to capsules where only alginate was used as a protective agent, positioning the *S. hispanica* seeds as a high-yield source for encapsulation. Lastly, the ionic gelation technique is simple and inexpensive, so the production of protective capsules for molecules with biological potential using CM with this technique could offer products with beneficial health effects at low cost. Since this work was conducted under in vitro conditions, the findings will need to be confirmed by in vivo and clinical studies. Furthermore, future research could delve deeper into the structural characterization of the matrix, the identification of peptides, and the modeling of controlled release. Finally, aspects related to industrial scalability and system stability must be evaluated for practical application.

## 4. Materials and Methods

### 4.1. Seeds and Chemicals

The *Salvia hispanica* and *Phaseolus lunatus* seeds were purchased from the main distributor in the area: Casa del Campesino, Merida, Yucatan, Mexico. The seeds were ground to obtain wholemeal flour using a Cemotec mill Tecator (Höganäs, Sweden) and subsequently finely ground using a Cyclotec 1093 mill Tecator (Höganäs, Sweden) until a flour with a particle size of 147 µm was obtained. Reagents were analytical-grade and purchased from J.T. Baker (Phillipsburg, NJ, USA), SIGMA (Sigma Chemical Co., St. Louis, MO, USA), and Merck (Darmstadt, Germany).

### 4.2. Chia Mucilage (CM) Extraction

The CM was obtained in accordance with the methodology proposed by Tavares et al. [42] with some modifications. A seed suspension was prepared using distilled water in a ratio of 1:20, stirring for 4 h at 60 °C. The mucilage was separated from the seeds by vacuum filtration using a 100 µm mesh at 220 mbar, then frozen at −20 °C for 96 h, and finally freeze-dried (−45 °C, 0.060 mbar) (LABCONCO freeze dryer, Freezone 18; Fort Scott, KS, USA).

### 4.3. PPC, Hydrolysis and Hydrolysis Degree

The PPC was obtained by wet fractionation of PF and isoelectric precipitation. A suspension of PF in distilled water was prepared in a 1:6 ratio (*w*/*v*), the pH was adjusted to 11 with 1 N NaOH, and it was stirred at 400 rpm for 1 h with a mechanical stirrer (Caframo RZ-1, Wiarton, ON, Canada). After this time, the fiber-rich fraction was removed, and the pH was adjusted to 4.5 with 1 N HCl to obtain the concentrate. Hydrolysis was performed following the technique proposed by Ciau-Solís et al. [43], where the hydrolysis parameters were 4% substrate, enzyme–substrate ratio 1:10 (*w*/*v*), temperature 37 °C, pH 2 for the reaction with pepsin (45 min), and pH 7.5 for the reaction with pancreatin (45 min), with mechanical agitation at 300 rpm (Caframo RZ-I). The degree of hydrolysis was then determined using the technique described by Nielsen et al. [44], calculating the ratio between free amino groups by reaction with o-phthalaldehyde (OPA) reagent using a serine standard. The total number of amino groups was determined in a sample 100% hydrolyzed with HCl at 110 °C for 24 h in a vacuum oven. The soluble fraction of the hydrolysate was subjected to ultrafiltration using 10 kDa cut-off membranes in an Amicon Model 2000 ultrafiltration unit (Millipore, Inc., Marlborough, MA, USA) to obtain a peptide fraction < 10 kDa. This fraction was dried by freeze-drying (−45 °C, 0.060 mbar). The protein content of the peptide fraction was determined using the technique proposed by Lowry [45]. A total of 0.1 mL of diluted sample was mixed with 5 mL of alkaline cupric reagent (2% Na_2_CO_3_ in 0.1 N NaOH, 0.5% CuSO_4_·5H_2_O, and 1% sodium–potassium tartrate) and incubated for 10 min at room temperature. Subsequently, 0.5 mL of Folin–Ciocalteu reagent (1:1 *v*:*v*) was added, mixed immediately, and allowed to develop color for 30 min in the dark. The absorbance was measured at 750 nm using a spectrophotometer (VELAB model VE-5100UV, Mexico City, Mexico), and the protein concentration was calculated using a standard curve prepared with bovine serum albumin (BSA) at concentrations ranging from 0 to 1 mg/mL.

### 4.4. Proximate Composition of Raw Materials

The proximal composition of the seed flour, the chia mucilage, and the protein concentrate obtained from *P. lunatus* was determined according to the methodologies proposed by the AOAC International [46] for nitrogen content (954.01 method), fat (920.39 method), ash (942.05 method), crude fiber (962.09 method), and moisture (925.09 method). Total carbohydrates were estimated by difference at 100% as a nitrogen-free extract (NFE).

### 4.5. Determination of ACE-I Activity

ACE-I activity was determined according to the methodology proposed by Hayakari et al. [47] in the PH and <10 kDa peptide fraction from *P. lunatus*. ACE from rabbit lung (A6778) was purchased from Sigma Aldrich 2U equal to 1 mg of protein) was reconstituted in milli-q water (1 mL) to produce the ACE stock solution (1 U/mL, 500 μg/mL of protein). This method is based on the colorimetric reaction of hippuric acid with 2,4,6-trichloro-s-triazine (TT). A hippuryl-l-histidyl-l-leucine (HHL) substrate was used at a 3% (*p*/*v*) concentration in 0.1 M phosphate-based buffers (pH 8.3) and a 5 M NaCl solution. Once assay time was complete, the reaction was stopped with TT in dioxane (3% *w*/*v*), and 5 min later the samples were centrifuged at 15,250× *g* for 10 min at 4 °C (Hermle Z300K model, Wehingen, Germany). Absorbance was read at 382 nm in a spectrophotometer VELAB model VE-5100UV. All runs were done in triplicate. The IC_50_ value (the peptide concentration required to produce 50% ACE inhibition) was quantified by a regression analysis of ACE-I activity (%) versus the peptide concentration (mg protein/mL).

### 4.6. Microencapsulation Design

The capsules formed with CM:Al were prepared according to the methodology proposed by Sandoval-Peraza et al. [22]. A 2^3^ factorial design with four central treatments was performed (Table 7). The response factors were the amount of protein released into the intestinal medium and residual ACE-I activity. A total of 1.5 g of the CM:Al combinations was dispersed in 150 mL of distilled water and 400 mg of the <10 kDa peptide fraction of *P. lunatus*. Once dissolved, the entire solution was passed through a peristaltic pump (Cole-Palmer, Model 7553-70, Barrington, IL, USA) with a Masterflex hose with a diameter of 2 mm and a pumping speed of 0.17 mL/s. The hose outlet was placed at a height of 10 cm from the beaker containing the CaCl_2_ solution (250 mL). Once the CM:Al dispersion and peptide fraction had been added, the hardening times specified in the design were followed. Finally, the capsules were obtained by decantation and washed with deionized water, then dried by freeze-drying. Additionally, a 100% control was performed with 0.15 M CaCl_2_ alginate and 30 min of hardening.

### 4.7. Encapsulation Efficiency and Morphology

The encapsulation efficiency was calculated according to Equation (1) as shown below:

(1) $$\mathrm{Efficiency}\;=\;\frac{\left(Protein\;in\;treatment-protein\;in\;control\right)\;\times \;Total\;weight\;of\;formed\;capsules}{Weight\;of\;peptide\;fraction\;in\;formulation}\;\times \;100\;$$

To determine the morphology and diameter, five capsules were taken at random and viewed using a stereoscope (5× MOTIC SMZ-168, Richmond, BC, Canada). The images were taken with the device’s 10 MP camera and processed with Motic-Images Manager software (V.Plus 2.0). The areas (mm^2^) were measured using ImageJ 1.47 software.

### 4.8. Calcium Uptake, Angle of Repose, and DSC

Calcium uptake was measured using the methodology reported by Suksamran [48]. Ten milliliters of CaCl_2_ solution was taken before and after the encapsulation process. The samples were neutralized with 1 M NaOH, 10 mg of hydroxynaphthol blue was added, and they were titrated with 0.05 M EDTA. Each determination was performed in triplicate.

The angle of repose was calculated according to the methodology proposed by Sankalia et al. [49]. A total of 10 g of capsules was passed through a tunnel (12 mm internal diameter, 70 mm length) 10 cm from a flat horizontal base to form a pile. Pile height (h) and cone base radius (r) were measured with a vernier, and the angle of repose (φ) was calculated with Equation (2). Fluency was rated according to the interval proposed by Shah et al. [20].

(2) $$\varphi =tan\;\frac{-1\;h}{r}\;$$

Differential scanning calorimetry (DSC) was performed following the methodology by Kaur et al. [50]. In total, 3 mg (db) of capsules were weighed out, and water was added until reaching a 20% (*w*/*w*) concentration. This mixture was crimped into a standard aluminum pan (Perkin-Elmer, No. 0219-0041) and heated from 30 to 200 °C at a 10 °C/min rate under constant nitrogen purging at 20 mL/min. Thermograms were generated using a calorimeter (DSC-6, Perkin-Elmer, Shelton, CT, USA). Blank capsules containing no peptide fraction were prepared using the conditions shown in Table 7.

### 4.9. In Vitro Release Studies, Protein Released, and Remaining ACE-I

For each treatment, in vitro release capacity was evaluated with an adapted version of the method by Takagi et al. [51]; 100 mg of dry capsules were placed into 50 mL beakers briefly, and then 25 mL of HCl solution at pH 2 with 2 mg/mL of NaCl was added. This mixture was shaken with a multi-position magnetic stirrer (Variomag Poly 15, 2mg AG, IL, USA) at 350 rpm for 2 h at 37 °C to simulate the gastric medium (GM). The microcapsules were recovered by decanting the solution and placed into a 50 mL beaker containing 25 mL of 0.25 M phosphate buffer at pH 6.8 and shaken at 1.5 rpm 3 h at 37 °C to simulate the intestinal medium (IM). The solutions of GM and IM simulations of each sample were stored in 50 mL conical centrifuge tubes for subsequent evaluation of released protein content by Lowry [45] and residual ACE-I activity according to Hayakari et al. [47].

### 4.10. Amino Acid Profiles by HPLC

The profile content of Asp + Asn, Glu + Gln, Ser, His, Gly, Thr, Arg, Ala, Pro, Tyr, Val, Met, Cys, Ile, Leu, Phe, and Lys was quantified according to the technique proposed by Alaiz et al. [52]. The amino acid profile of peptide fraction < 10 kDa and released peptides in intestinal digestion were determined by reversed-phase high-performance liquid chromatography (HPLC). The spectrophotometric detection was measured at 280 nm. The samples were hydrolyzed with 6 N HCl and dried in a vacuum oven. Derivatization was done with diethylethoxymethylenemalonate for 45 min at 50 °C. The equipment used was an Agilent HP 1100 series with automatic injection and a VWD detector. The technique required DL-α-aminobutyric acid as an internal standard and a C18 reverse-phase chromatographic column (Nova Pack brand, particle size 4 μm, 300 × 3.9 mm). The resolution was performed using a binary gradient system: 25 mM sodium acetate solution with 0.02% sodium azide (pH 6) and acetonitrile with a constant flow of 0.9 mL/min at 18 °C. Trp was determined according to Yust et al. [53] using basic hydrolysis (NaOH 4 N) and diluted in sodium borate buffer (pH 9). An isocratic elution system consisting of 25 mM sodium acetate, 0.02% sodium azide (pH 6), and acetonitrile (91:9) delivered at 0.9 mL/min, with the same equipment, column, and temperature described above, was used.

### 4.11. Statistical Analysis

The results of the experiments were performed in triplicate (n = 3) and processed using descriptive statistics with measures of central tendency and dispersion. Analysis of variance and regression were performed for each experiment, corresponding to the 2^3^ factorial design, with 4 central-point replicates to identify differences in each response variable and their optimal conditions. Subsequently, the optimal conditions for the encapsulation process were determined using a multiple response analysis with a desirability function. The statistical power (1 − β) of the design was calculated considering the encapsulation efficiency and ACE-inhibitory activity, taking as a reference the range observed between the maximum and minimum values. The resulting Cohen’s *f* values were 1.92 and 2.10, respectively [54], yielding an estimated statistical power well above the 80% threshold and confirming the sensitivity of the design. All analyses were performed following Montgomery [55], using Statgraphics Centurion version 19 software (Statgraphics Technologies, Inc., The Plains, VA, USA).

## 5. Conclusions

This study confirms the hypothesis that the incorporation of chia mucilage (*Salvia hispanica*), combined with sodium alginate, constitutes an effective encapsulation matrix for protecting ACE-inhibiting peptides (<10 kDa) from *Phaseolus lunatus* hydrolysates during simulated gastrointestinal digestion. The optimized formulation (CM:Al ratio 30:70; 0.05 M CaCl_2_; 20 min hardening) achieved an encapsulation efficiency of 47.78% and preserved bioactivity with IC_50_ values of 0.1 mg/mL after simulated digestion, significantly higher than pure alginate systems. It is important to note that the ACE-I activity reported in this study was determined under in vitro conditions, and as documented in the literature, in vitro ACE-I does not necessarily predict in vivo antihypertensive efficacy. Therefore, these results should be interpreted as evidence of in vitro bioactive potential, and future in vivo studies will be required to establish actual antihypertensive efficacy.

Overall, the results highlight the potential of the comprehensive use of plant resources such as *Salvia hispanica* and *Phaseolus lunatus* for the development of sustainable functional systems, positioning chia mucilage as an effective plant-based encapsulating agent for the protection of bioactive compounds with bioactive and possible physiological applications. This work provides a scientific basis for the use of regional biopolymers in the design of functional capsules capable of withstanding industrial thermal processes without compromising their functional efficacy.

## Figures and Tables

**Table 1:** Proximal composition of raw materials (% db, except moisture).

Component	CF	CM	PF	PPC
Moisture	7.95 ^a^ ± 0.00	6.94 ^b^ ± 0.18	12.71 ^A^ ± 0.09	2.80 ^B^ ± 0.15
Protein	28.16 ^a^ ± 0.45	6.51 ^b^ ± 0.01	21.98 ^B^ ± 0.01	70.36 ^A^ ± 0.27
Crude fiber	24.97 ^a^ ± 0.40	16.46 ^b^ ± 0.41	4.44 ^A^ ± 0.25	0.35 ^B^ ± 0.02
Fat	25.06 ^a^ ± 0.43	0.83 ^b^ ± 0.06	3.23 ^A^ ± 0.20	3.63 ^A^ ± 0.18
Ash	4.35 ^b^ ± 0.08	8.07 ^a^ ± 0.22	4.04 ^A^ ± 0.02	4.15 ^A^ ± 0.09
Carbohydrates as NFE *	17.44 ^b^ ± 0.58	68.13 ^a^ ± 0.55	66.29 ^A^ ± 0.4	21.48 ^B^ ± 0.35

All analyses were done in triplicate. The data represents the average value ± standard error. ^a,b/A,B^ Superscripts in the same row indicate significant statistical differences (*p* < 0.05) for chia or *P. lunatus*, respectively. * NFE was calculated by difference.

**Table 2:** Morphology and areas of wet and dry capsules.

Treatment	(A)(B)(C)	Control	Dry Control	Treatment	Dry Treatment
1	(−)(−)(−)	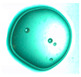 12.59 mm^2^	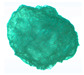 5.05 mm^2^	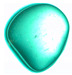 10.55 mm^2^	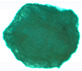 5.37 mm^2^
2	(+)(−)(−)	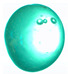 8.78 mm^2^	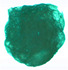 4.5 mm^2^	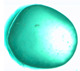 7.2 mm^2^	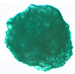 5.15 mm^2^
3	(−)(+)(−)	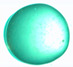 10.28 mm^2^	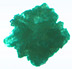 2.7 mm^2^	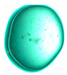 8.97 mm^2^	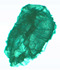 5.97 mm^2^
4	(−)(−)(+)	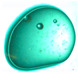 9.61 mm^2^	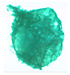 5.1 mm^2^	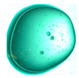 10.11 mm^2^	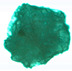 5.77 mm^2^
5	(+)(+)(−)	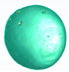 7.41 mm^2^	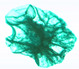 1.37 mm^2^	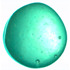 7.36 mm^2^	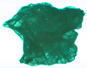 2.1 mm^2^
6	(+)(−)(+)	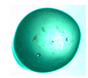 6.99 mm^2^	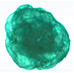 3.57 mm^2^	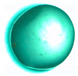 8.19 mm^2^	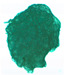 2.82 mm^2^
7	(−)(+)(+)	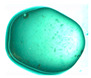 8.28 mm^2^	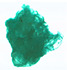 1.66 mm^2^	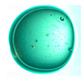 11.6 mm^2^	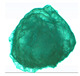 4.3 mm^2^
8	(+)(+)(+)	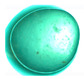 6.37 mm^2^	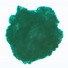 1.51 mm^2^	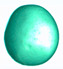 6.29 mm^2^	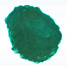 2.59 mm^2^
9–12	(0)(0)(0)	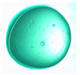 7.84 mm^2^	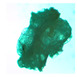 8.31 mm^2^	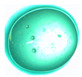 7.86 mm^2^	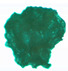 7.45 mm^2^
Alginate (Al)	(Al)(+)(+)	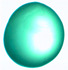 4.6 mm^2^	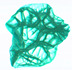 1.25 mm^2^	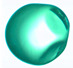 3.83 mm^2^	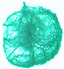 1.29 mm^2^

A: (+) CM:Al (70:30); (*−*) CM:Al (30:70); (0) CM:Al (50:50) blend ratio. B: (+) 0.15; (*−*) 0.05; (0) 0.1 M of CaCl_2_. C: (+) 30 min; (*−*) 20 min; (0) 25 min of hardening time. Al: 100% alginate.

**Table 3:** Total protein and encapsulation efficiency (%) of peptide fraction < 10 kDa from *P. lunatus* in microcapsules with CM:Al.

Treatment	(A)(B)(C)	Total Protein (%)	Encapsulation Efficiency (%)
1	(−)(−)(−)	12.46 ± 0.15	47.78 ^c^ ± 0.57
2	(+)(−)(−)	9.51 ± 0.11	35.12 ^a,b^ ± 0.39
3	(−)(+)(−)	8.58 ± 0.31	35.84 ^a,b^ ± 1.30
4	(−)(−)(+)	9.27 ± 0.66	32.17 ^a,b^ ± 1.12
5	(+)(+)(−)	8.56 ± 0.56	37.04 ^b^ ± 1.34
6	(+)(−)(+)	8.87 ± 0.1	29.51 ^a^ ± 0.33
7	(−)(+)(+)	7.70 ± 0.16	34.31 ^a,b^ ± 0.73
8	(+)(+)(+)	11.01 ± 0.08	46.19 ^c^ ± 0.36
9–12	(0)(0)(0)	8.29 ± 0.28	33.23 ^a,b^ ± 2.43
Alginate (Al)	(Al)(+)(+)	5.82 ± 0.08	21.05 * ± 0.28

A: (+) CM:Al (70:30); (*−*) CM:Al (30:70); (0) CM:Al (50:50) blend ratio. B: (+) 0.15; (*−*) 0.05; (0) 0.1 M of CaCl_2_. C: (+) 30 min; (*−*) 20 min; (0) 25 min of hardening time. Al: 100% alginate. * Statistically different (*p* < 0.05) compared to the treatments. ^a–c^ Superscripts in the same column indicate significant statistical differences (*p* < 0.05)

**Table 4:** Calcium uptake, angle of repose, and thermal transition of CM and alginate capsules with the peptide fraction of *P. lunatus* < 10 kDa.

Treatment	(A)(B)(C)	Calcium Controls	Calcium Treatments	Angle of Repose	Thermal Transition Control	Thermal Transition Treatments
1	(−)(−)(−)	38.61 ^c,d^ ± 0.90	45.89 ^b^ ± 0.45	35.99 ^c^ ± 1.93	175.57 ^a^	178.68 ^a^
2	(+)(−)(−)	45.89 ^e^ ± 0.45	50.00 ^c^ ± 0.90	34.45 ^b,c^ ± 1.00	175.95 ^a^	177.21 ^a^
3	(−)(+)(−)	38.78 ^c,d^ ± 0.32	40.68 ^a^ ± 0.96	42.86 ^d^ ± 2.08	170.29 ^a^	178.63 ^a^
4	(−)(−)(+)	36.08 ^b,c^ ± 0.90	44.30 ^b^ ± 0.00	35.16 ^c^ ± 1.23	174.28 ^a^	175.57 ^a^
5	(+)(+)(−)	38.20 ^c^ ± 0.64	48.80 ^c^ ± 0.96	34.44 ^b,c^ ± 1.79	177.5 ^a^	178.35 ^a^
6	(+)(−)(+)	45.89 ^e^ ± 0.45	50.63 ^c^ ± 0.00	31.53 ^b^ ± 1.43	175.45 ^a^	175.99 ^a^
7	(−)(+)(+)	34.36 ^b^ ± 0.32	38.87 ^a^ ± 0.32	36.76 ^c^ ± 1.23	176.57 ^a^	178.50 ^a^
8	(+)(+)(+)	41.13 ^d^ ± 0.32	45.64 ^b^ ± 0.32	25.89 ^a^ ± 2.04	168.45 ^a^	176.9 ^a^
9–12	(0)(0)(0)	29.91 ^a^ ± 0.98	44.72 ^b^ ± 0.91	36.66 ^c^ ± 1.33	172.8 ^a^	174.02 ^a^
Alg (Al)	(Al)(+)(+)	37.07 ^a^ ± 0.32	56.92 * ± 0.32	35.08 * ± 0.90	174.02 ^a^	174.43 ^a^

A: (+) CM:Al (70:30); (*−*) CM:Al (30:70); (0) CM:Al (50:50) blend ratio. B: (+) 0.15; (*−*) 0.05; (0) 0.1 M of CaCl_2_. C: (+) 30 min; (*−*) 20 min; (0) 25 min of hardening time. Al: 100% alginate. ^a–e^ Superscripts in the same column indicate significant statistical differences (*p* < 0.05). * Statistically different (*p* < 0.05) compared to the treatments.

**Table 5:** Peptide fraction released (mg) in gastric (GM) and intestinal (IM) mediums and its ACE-I activity (IC_50_).

Treatment	(A)(B)(C)	Protein Released in Gastric Medium (mg)	Protein Released in Intestinal Medium (mg)	ACE-I (IC_50_ mg/mL) GM	ACE-I (IC_50_ mg/mL) IM
1	(−)(−)(−)	16.96 ^c,d,e^ ± 0.20	6.40 ^b,c^ ± 0.30	0.25 ^d,e^	0.10 ^b^
2	(+)(−)(−)	15.16 ^b,c^ ± 0.91	5.61 ^a,b^ ± 0.41	0.22 ^b,c^	0.09 ^b^
3	(−)(+)(−)	22.64 ^f^ ± 0.71	3.81 ^a^ ± 0.30	0.33 ^g^	0.06 ^a^
4	(−)(−)(+)	16.24 ^c,d^ ± 0.20	7.90 ^c^ ± 0.20	0.24 ^c,d^	0.13 ^c^
5	(+)(+)(−)	19.26 ^e^ ± 0.20	4.24 ^a^ ± 0.51	0.28 ^f^	0.07 ^a^
6	(+)(−)(+)	13.87 ^b^ ± 0.30	5.61 ^a,b^ ± 0.41	0.20 ^b^	0.09 ^b^
7	(−)(+)(+)	16.67 ^c,d^ ± 0.20	5.61 ^a,b^ ± 0.20	0.25 ^d,e^	0.09 ^b^
8	(+)(+)(+)	17.82 ^d,e^ ± 0.00	3.81 ^a^ ± 0.51	0.26 ^e,f^	0.06 ^a^
9–12	(0)(0)(0)	10.53 ^a^ ± 0.84	6.31 ^b^ ± 0.67	0.15 ^a^	0.10 ^g^
Alg (Al)	(Al)(+)(+)	16.49 ± 0.08	0.00 * ± 0.00	0.24 *	0.00 *

A: (+) CM:Al (70:30); (*−*) CM:Al (30:70); (0) CM:Al (50:50) blend ratio. B: (+) 0.15; (*−*) 0.05; (0) 0.1 M of CaCl_2_. C: (+) 30 min; (*−*) 20 min; (0) 25 min of hardening time. Al: 100% alginate. * Statistically different (*p* < 0.05) compared to the treatments. ^a–g^ Superscripts in the same column indicate significant statistical differences (*p* < 0.05).

**Table 6:** Amino acid profile in the peptide fraction < 10 kDa and fraction released by each treatment in intestinal medium (g/100 g of protein).

Amino Acid	<10 kDa	T 1	T 2	T 3	T 4	T 5	T 6	T 7	T 8	T 9–12
Asp + Asn	10.83	11.59	12.06	11.24	12.34	12.14	12.28	12.14	12.17	11.40
Glu + Gln	12.48	13.29	14.53	13.45	14.51	13.42	14.88	14.29	14.36	12.91
Ser	4.63	4.82	5.70	4.65	5.92	5.75	5.91	5.70	5.60	5.29
His	1.89	2.30	2.99	2.48	1.74	2.28	3.21	2.01	2.35	2.31
Gly	5.27	5.70	5.64	6.48	6.45	5.19	5.86	5.63	5.51	5.50
Thr	4.03	4.02	4.52	4.32	4.01	3.96	4.49	4.00	4.22	4.11
Arg	11.10	11.45	10.30	12.04	10.89	9.43	10.34	10.09	9.86	10.42
Ala	2.16	2.60	2.68	2.66	2.54	2.32	2.82	2.23	2.27	2.33
Pro	9.35	8.70	6.91	4.24	9.87	14.95	5.86	12.23	12.54	12.05
Tyr	3.49	2.39	2.44	3.11	1.85	1.75	2.18	1.66	1.73	2.21
Val	5.37	5.92	5.76	6.20	5.44	5.27	5.63	5.51	5.42	5.71
Met	0.06	0.79	0.64	1.14	0.09	0.07	0.77	0.08	0.10	0.46
Cys	0.71	DNQ	DNQ	DNQ	DNQ	DNQ	DNQ	DNQ	DNQ	DNQ
Ile	4.70	5.03	4.90	5.35	4.57	4.47	4.74	4.57	4.56	4.81
Leu	9.18	8.56	7.88	9.18	7.89	7.31	7.79	7.92	7.59	8.12
Phe	6.02	5.36	5.08	5.97	4.55	4.46	5.04	4.73	4.57	5.00
Lys	6.40	7.48	7.96	7.47	7.34	7.22	8.21	7.21	7.16	7.37
Trp	2.34	DNQ	DNQ	DNQ	DNQ	DNQ	DNQ	DNQ	DNQ	DNQ

DNQ: detectable but not quantifiable.

**Table 7:** Encapsulation conditions of capsules produced using CM:Al blends, CaCl_2_ as an ion bond, and hardening time.

Treatment	Levels	CM:Al Ratio (A)	CaCl_2_ M (B)	Hardening Time (C)
1	(−)(−)(−)	30:70	0.05	20
2	(+)(−)(−)	70:30	0.05	20
3	(−)(+)(−)	30:70	0.15	20
4	(−)(−)(+)	30:70	0.05	30
5	(+)(+)(−)	70:30	0.15	20
6	(+)(−)(+)	70:30	0.05	30
7	(−)(+)(+)	30:70	0.15	30
8	(+)(+)(+)	70:30	0.15	30
9–12 Central treatments	(0)(0)(0)	50:50	0.1	25
Alginate (Al)	(Al)(+)(+)	100	0.15	30

(−) lowest, (+) highest, and (0) central levels of experimental design. Al, corresponds to the use of alginate only.

## Data Availability

The data presented in this study are available on request from the corresponding author due to privacy restrictions.

## Abbreviations

The following abbreviations are used in this manuscript:

CF	Chia flour
CM	Chia mucilage
PF	*Phaseolus lunatus* flour
PH	*Phaseolus lunatus* hydrolysate
PPC	*Phaseolus lunatus* protein concentrate
NFE	Nitrogen-free extract
ACE-I	Angiotensin-converting enzyme inhibition
Al	Sodium alginate
DSC	Differential scanning calorimetry
